# Predictors of self-rated health in older adults: insights from an anchoring vignettes study

**DOI:** 10.1007/s10433-026-00952-7

**Published:** 2026-10-06

**Authors:** Karolina Thune, Hanna Falk Erhag, Anne Ingeborg Berg, Lena Johansson, Maria Solevid

**Affiliations:** 1 Institute for Health and Care Science, Sahlgrenska Academy, University of Gothenburg, Gothenburg, Sweden; 2 Department of Acute Medicine and Geriatrics, Sahlgrenska University Hospital, Region Västra Götaland, Gothenburg, Sweden; 3 Department of Psychology, University of Gothenburg, Gothenburg, Sweden; 4 Department of Addiction Disorders, Sahlgrenska University Hospital, Region Västra Götaland, Gothenburg, Sweden; 5 Department of Political Science, University of Gothenburg, Gothenburg, Sweden

**Keywords:** Subjective health, Health trajectories, Sex differences, Survey data

## Abstract

Self-rated health is one of the most used measures of subjective health in research. At the same time, it presents several methodological challenges. Self-rated health requires individuals to translate their complex, multifaceted health experiences into a single response on the self-rated health scale. When different groups interpret and rate their health differently e.g. by gender or birth cohorts, the risk of misinterpretation increases. Older adults tend to rate their health as good or very good despite the increasing physical degeneration associated with ageing. To address these challenges, the anchoring vignette method was developed to improve the comparability of survey responses. In this study, anchoring vignettes were used to investigate the association between demographic and health-related predictors and both unadjusted and adjusted self-rated health in later life. Data were collected through the Swedish Citizen Panel; a total of 4,300 individuals aged 60–85 were invited, stratified by age and sex. 3,083 participants completed more than 80% of the questionnaire. The associations between demographic and health-related factors and self-rated health changed between the unadjusted and adjusted analyses. In the unadjusted analysis, women reported better health than men; this reversed in the CHOPIT model. After adjustment, there was no significant association between self-rated health and age or educational level. Living with others remained positively associated with self-rated health, whereas dependency in daily activities, number of diseases, and somatic symptoms remained negatively associated. We conclude that anchoring vignettes can be a suitable tool for evaluating self-rated health in later life and a valuable addition to simpler, traditional approaches.

**Supplementary Information:**

The online version contains supplementary material available at https://doi.org/10.1007/s10433-026-00952-7.

## Introduction

Self-rated health (SRH) is a widely used, yet methodologically challenging measure that can facilitate understanding, track change, predict outcomes, and guide interventions (Idler & Benyamini 1997; Jylhä 2009). SRH is commonly defined as a subjective, individual-level measure of overall health status, comprising multiple interacting dimensions that unfold over time. It is typically assessed using a single-item question, such as *“In general, how would you rate your health?”* To answer, individuals translate their “qualia” (i.e. the felt quality of experience—in this case, how health *feels*) into a single response on a rating scale ranging from *“excellent”* to *“poor”* (Jylhä 2009; De Salvo et al. 2006; Spuling et al. 2015; Idler & Benyamini 1997). Its predictive ability and pragmatic strengths make it a compelling measure in research, and numerous studies have demonstrated that lower SRH is associated with increased morbidity and mortality independent of other health parameters, particularly among older adults (Whitmore et al. 2022; Jylhä 2009; DeSalvo et al. 2006; Idler & Benyamini 1997). As such, SRH captures meaningful variance in health beyond medically defined objective health status by reducing a complex, subjective experience into a single rating. This process requires individuals to function as both observers and interpreters of their own health. Consequently, two individuals with comparable objective health status might rate their health differently as a result of divergent internal reference points. When respondents interpret survey questions and apply response options inconsistently, the likelihood of such interpretive variation increases, thereby undermining the comparability of findings across individuals and groups.

Empirical studies support the notion that SRH responses are shaped by reference-group effects, in which individuals unconsciously assess their health relative to peers or prevailing community norms (Galenkamp et al 2013; Jylhä 2009; Spuling et al 2015). Such reference bias can obscure genuine health disparities. Older adults may under-report health problems due to adaptation and normalisation of age-related decline, while younger individuals may over-report minor symptoms. Self-assessments of health among older adults pose specific methodological challenges due to age-related changes, cohort differences, and survivor effects (Idler 1993). Different birth cohorts exhibit varying patterns of health perceptions, shaped by historical and social experiences (Heikkinen et al 2011; Idler 1993; Kekäläinen et al 2024; Schaap et al 2024; Spuling et al 2015).

To address these methodological challenges, King et al. (2003) developed the anchoring vignette method to increase the comparability of survey responses across individuals and groups (King et al. 2003; King and Wand 2007). In the context of SRH research, anchoring vignettes consist of brief hypothetical health scenarios. By asking respondents to rate the health of these sex- and age-matched scenarios in addition to their own health, the method enables researchers to identify and adjust for systematic differences in how individuals interpret and apply response scales. In doing so, anchoring vignettes offer a means of recalibrating SRH ratings, potentially improving both their comparability and analytical utility (Jürges and Winter 2013; Salomon et al 2004). Against this backdrop, the present study aims to investigate the association between demographic and health-related predictors and both unadjusted and adjusted SRH in a Swedish population of older adults aged 60–85.

### SRH and its response patterns in older adults

The ageing process typically entails deterioration in physical and functional health (Aarts et al. 2012; Barnett et al. 2012). Yet, despite the increasing prevalence of health challenges and functional impairments, older adults' SRH tends to remain relatively positive (Feenstra et al 2020). This apparent disconnect between objective health decline and subjective health appraisal has been termed the health-and-well-being paradox (Withmore 2022; Hansen and Blekesaune 2022). The paradox is thought to arise, because individuals adapt their internal standards as they age through complex psychological mechanisms that shape their health perceptions, for example, by recalibrating internal standards of comparison, shifting attention towards retained capabilities, or evaluating their health relative to age-matched peers rather than to their younger selves (Jylhä et al. 2001; Jylhä 2009; Leinonen et al. 2001). Consequently, although poor SRH is associated with chronic illness, functional disability, and mental health problems, and good SRH with favourable physical, functional, and mental health, these associations tend to attenuate with age (Galenkamp et al. 2013). Older adults are, therefore, a particularly relevant population for SRH research. Compared with younger adults, their self-ratings are to a greater extent shaped by reference-group effects, whereby individuals unconsciously assess their health relative to peers or prevailing community norms (Galenkamp et al. 2013; Jürges 2007; Jylhä 2009; Spuling et al. 2015, 2017). Such reference bias can obscure genuine health disparities.

Women generally report poorer SRH than men (Benyamini et al. 2000; Feenstra et al. 2020; Zajacova et al. 2017). They also experience more years living with disability, placing older women in a comparatively disadvantaged position (Wheaton & Crimmins 2016), despite their higher life expectancy relative to men, a phenomenon known as the male–female health-survival paradox (Philips et al. 2023). The observed differences between men's and women's SRH may partly reflect differing standards for appraising health, creating systematic sex differences in SRH (Benyamini et al. 2000). Specifically, women appear to base their SRH on a broader range of both health-related and non-health-related factors than men do. Women also tend to be more reflective in their health ratings and to consider a wider array of health dimensions, whereas men may be more influenced by stereotypes of masculine stoicism (Phillips et al. 2023).

### Identifying SRH reporting heterogeneity with anchoring vignettes

Despite its inherent ambiguity, SRH consistently predicts all-cause mortality even after adjusting for objective health measures, socioeconomic factors, and established risk factors (DeSalvo et al. 2006; Idler & Benyamini 1997). When SRH is compared across diverse populations, differences in how the question and its response options are interpreted may cause the item to function differently across groups, a phenomenon known as differential item functioning (DIF). DIF arises when individuals with equivalent levels of the underlying construct, in this study, health, have different probabilities of selecting particular response categories as a result of group membership rather than genuine differences in health (King et al. 2003; King & Wand 2007). For example, much of the apparent sex gap in SRH may stem from differences in reporting style, with evidence suggesting that older women tend to apply a slightly more “optimistic” benchmark when rating their own health (Grol-Prokopczyk et al. 2011).

Anchoring vignettes enhance the comparability of SRH across individuals and groups by accounting for systematic variation in interpretation. In doing so, they enable researchers to more effectively distinguish differences in health status from artefacts introduced by inconsistent reporting behaviour (Jürges & Winter 2013; Salomon et al. 2004). Beyond improving comparability, anchoring vignettes contribute to methodological rigour by reducing measurement error, increasing statistical power, and enhancing analytical precision (d'Uva et al. 2011; Datta Gupta 2010). The method has also proven useful for identifying response heterogeneity by age, education, socioeconomic status (SES), and sex (Hirve 2014).

### Adjusted and unadjusted SRH in older adults

Previous research has used anchoring vignettes to compare adjusted and unadjusted SRH and similar self-rating measures (Dowd & Todd 2011; Grol-Prokopczyk et al. 2011; Jürges & Winter 2013). A general conclusion drawn from these studies is that, once reporting differences are accounted for, the estimated effects of various predictors of SRH differ compared to the unadjusted ratings. Several specific findings illustrate this pattern. Respondents who received vignettes describing an individual older than themselves, signalled by the vignette characters name, tended to overstate their own health, whereas the opposite was observed among respondents who received vignettes describing a younger individual (Jürges & Winter 2013). Dowd & Todd (2011) used anchoring vignettes to test and adjust for reporting differences by educational level, ethnicity, and sex across six self-rated health domains. Although reporting differences were identified across all health domains, their magnitude varied by health domain and demographic characteristics. Notably, respondents provided more optimistic ratings with increasing age, a relationship that persisted across most domains even after adjustment. Grol-Prokopczyk et al. (2011) similarly found that, before adjustment, women and more highly educated individuals reported greater health optimism, whereas older respondents rated their health more poorly than younger respondents. After adjustment, the sex and age differences disappeared, and the positive effect of education attenuated, remaining significant only among respondents with a college degree.

In summary, anchoring vignettes are a potentially useful and valuable tool for analysing differences in SRH in later life, complementing traditional SRH measures by accounting for reporting heterogeneity. Studying response patterns in older adults is particularly relevant as SRH remains relatively stable with increasing age despite an increasing level of health disparities, such as chronic illnesses and disability. Consequently, it is not always clear whether observed differences in SRH between demographic groups reflect actual differences in health or differences in how individuals interpret and use response categories. Given that relatively few studies have used anchoring vignettes to examine SRH among older adults across age groups, this study contributes to the existing research on SRH and can provide a better understanding of how reporting behaviour influences older adults' SRH.

## Methods

### Design

To address potential DIF and better understand and correct for differences in self-ratings across groups of older adults, the anchoring vignette method was employed (King et al. 2003; King & Wand 2007). We developed four vignettes, informed by the general health vignettes designed by Grol-Prokopczyk et al. (2011). In anchoring vignette studies, vignettes are positioned between the response options of the self-ratings question; because SRH was measured on a five-point scale in the present study, the design entailed the use of four vignettes. To ensure relevance to the study population, the vignettes were tailored to older adults and described general aspects of health, such as fatigue, pain, and enjoyment of life, without reference to specific diseases. According to Grol-Prokopczyk (2017), general vignettes are less likely than disease-specific vignettes to violate the assumption of vignette equivalency (VE), that participants understand the vignettes similarly, and response consistency (RC), that participants apply the response categories consistently when rating both themselves and the vignettes. The vignette in the present study described different levels of health, ranging from no health impairments to more severe health conditions. Each respondent was presented with all four vignettes in randomised order. Translated versions of the vignettes are presented in Table 1. The names of the vignettes' characters were common Swedish names from the 1930s to the 1960s, selected with the target population in mind. According to Jürges & Winter (2013), cohort- and context-specific first names enhance respondents’ identification with the vignette character. (Jürges & Winter 2013). Participants were instructed to rate the vignette character as if they were the same age as themselves. All vignettes were sex-matched: male respondents received vignettes with male names, and female respondents received vignettes with female names, in line with established guidelines for anchoring vignette studies (King et al. 2003; King & Wand 2007).

**Table 1:** English translation of the vignettes used in this study

Previously, we asked you some questions about your health, where you rated how you feel today. Now, we are interested in how you would use the same response options to evaluate the health of others your age. You will read descriptions of individuals of your own age and their health status. After each description, you will be asked to rate their health using the same response options you used to rate your own health	
Very good/ Good	[Peter/Ingrid] experiences great enjoyment of life and has plenty of energy to devote to personal interests. [He/She] manages daily life without any difficulties and experiences no physical pain. [Peter/Ingrid] has not been sick during the past 3 months
Good/Fair	[Göran/Kristina] enjoys life and frequently has the energy to engage in personal interests. While [he/she] is generally able to manage daily activities independently, occasional assistance is needed with certain tasks. [Göran/Kristina] sometimes feels physical pain, though it does not significantly interfere with daily life. Over the past 3 months, [he/she] has been sick and confined to bed for 1 day
Fair/Poor	[Sven/Elisabeth] sometimes feels tired and sad, which leaves [him/her] with little energy for personal interests. [He/She] has difficulty managing daily life independently and frequently requires assistance. [Sven/Elisabeth] occasionally experiences physical pain that affects both daily life and overall enjoyment. During the past 3 months, [he/she] has been sick several times and has sought medical care, but has not been hospitalised
Poor/Very poor	[Anders/Birgitta] often feels tired and sad and lacks the motivation to engage in personal interests. [He/She] has significant difficulty managing daily life independently and requires personal assistance. [Anders/Birgitta] experiences severe physical pain every day, which negatively affects both daily life and overall zest for life. During the past 3 months, [he/she] has required hospitaliation for a few days and has been sick and bedridden at home for an entire week

Note: English translation of the original vignettes in Swedish

### Data collection

The present study followed the procedure described by King & Wand (2007). Participants first rated their own SRH and answered sociodemographic and health-related questions. Subsequently, they rated the health of the vignette characters. Data were collected through the Swedish Citizen Panel (Swedish: Medborgarpanelen), administered by the SOM (Society Opinion Media) Institute, an independent survey research organisation, at the University of Gothenburg, in Gothenburg, Sweden. The panel comprises approximately 75,000 pre-recruited panellists drawn from both self-recruited and probability-based samples, with approximately 25% recruited through probability-based sampling. Of the probability-based panellists, approximately 7,000 are older than 60 years. The present study drew on the probability-based sample, with data collected between 4 December 2024 and 7 January 2025 (Bergström et al. 2024). A total of 4300 panellists were invited, stratified on age and sex (see Supplementary Table 1). Of these, 3083 completed more than 80% of the questionnaire, and 2944 (68.5%) provided complete responses to all variables included in the present analyses, including SRH, sex, age, education, cohabitation status, activities of daily living (ADL), instrumental activities of daily living (IADL) number of diseases, and the Somatic Symptom Scale−8 questionnaire (SSS-8) (Gierket et al. 2014). In preparation for this study, a pilot study was conducted in December 2023 using a self-recruited sample of 4,300 individuals aged 70–90 years from the Swedish Citizen Panel. The pilot identified areas for improvement, which informed the subsequent inclusion of the somatic symptom burden scale. Importantly, the central findings were consistent across both studies. Results of the pilot study are available from the first author upon request.

### Measures

SRH, the primary outcome variable in this study, was assessed using the single-item question “In general, how would you rate your health?” with five response options ranging from *very poor* to *very good.* Sex was recorded as man or woman. Age was categorised into five groups: 60–64, 65–69, 70–74, 75–79, and 80–85 years. Educational level was classified into four categories: primary education or less, upper secondary education, post-secondary education (without a university degree), and university degree. Cohabitation status was dichotomised as cohabiting or living alone. Somatic symptom burden was measured using the SSS-8 (Gierk et al. 2014), which assesses eight symptoms: back pain; chest pain or shortness of breath; dizziness; feeling tired or having low energy; headaches; pain in arms, legs, or joints; stomach or bowel problems; and trouble sleeping. Each symptom was rated on a five-point scale from 0 (not at all) to 4 (very much), yielding a total score between 0 and 32. In line with Gierk et al., (2014), the total score was categorised into five levels of symptom burden: no to minimal (0–3 points), low (4–7 points), medium (8–11 points), high (12–15 points), and very high (16–32 points). The ADL and IADL items comprised 13 activities, ranging from basic personal activities (e.g. bathing, toileting, transferring), to more complex activities (e.g. cleaning, preparing meals, managing medications). For each activity, respondents indicated whether they performed it independently (with or without aids) or required assistance from another person. Respondents reporting a need for help with one or more activities were classified as ADL/IADL dependent (see Supplementary Table 2). The number of chronic diseases was assessed by asking participants whether, within the past 10 years, they had been told by a physician or nurse that they had any of the following conditions: ‘cardiovascular disease, haematological disease, pulmonary disease, eye or ear disorder, gastrointestinal disease, diseases of the internal organs, genitourinary or reproductive disorder, musculoskeletal or skin disease, neurological disease, endocrine or metabolic disease, psychiatric disorder, and dementia’. The number of reported conditions was grouped into three categories: 0–1, 2–3, and 4 or more. ADL/IADL limitations and chronic diseases were entered as categorical variables, following established operationalizations in previous studies (Clark et al. 2015; Falk Erhag et al., 2021; Thörnqvist et al. 2025), to avoid imposing a linear association between these grouped health indicators and SRH.

### Analysis

The analytical strategy comprised several complementary methods. First, descriptive analyses summarised sample characteristics and the distribution of SRH and vignette ratings. To examine the predictors of correct vignette ordering, we estimated a linear probability model (LPM) and a logit model. The associations between sociodemographic and health-related variables and vignette ratings were then assessed using ordinary least squares (OLS) regression.

Subsequently, ordered probit (OPROBIT) and compound hierarchical ordinal probit (CHOPIT) models were used to examine the associations sociodemographic and health-related factors with unadjusted and adjusted SRH. Sex, age, educational level, cohabitation status, ADL/IADL dependency, somatic symptom burden, and number of diseases were included as covariates in all analyses unless otherwise specified. In the first set of models, OPROBIT was applied to both the unadjusted SRH and non-parametrically adjusted (i.e. manually) SRH measure. The non-parametric adjustment was restricted to respondents who ranked the vignettes in the expected order (vignette 1 > vignette 2 > vignette 3 >vignette 4), and respondents who did not rank the vignettes in this order were excluded from this adjustment, thereby ensuring consistent benchmarks (King et al. 2003; King & Wand 2007). The adjustment compared each respondent’s SRH with the position of their vignette ratings and rescaled the original five-point measure to a nine-point scale, reflecting the respondent's position relative to the vignette thresholds. In the second set of analyses, the CHOPIT method was used to jointly model respondents’ SRH and their vignette evaluations (King & Wand 2007). In contrast to the non-parametric approach, CHOPIT neither excludes respondents with misordered vignette ratings nor rescales the outcome. Instead, all respondents with valid responses on the included variables were retained in the analysis. The model estimates how covariates influence both the hidden level of health (i.e. the latent level that we want to measure) and the thresholds that map this latent health onto the survey’s five-point response scale. The covariate coefficients represent the effects on the latent level of health, whereas the threshold coefficients capture systematic differences in the use of response categories. CHOPIT can, thus, accommodate group-specific cut points by anchoring respondents’ self-ratings to their vignette ratings (Grol-Prokopczyk 2014; Grol-Prokopczyk et al. 2011), thereby enabling genuine health differences to be distinguished from reporting behaviour. For these reasons, the CHOPIT model results constitute the primary basis for our conclusions. As a final step, however, these results were compared with those from the OPROBIT model using unadjusted SRH.

## Results

The participants' characteristics are presented in Table 2. Most participants reported favourable SRH: 52.1% reported Good SRH, and 24.7% reported Very Good SRH. The sex distribution was nearly equal, with 49.3% women and 50.7% men. The mean age was 71 years (range, 60–85). The sample was highly educated; 25% reported post-secondary education, and 41% reported having a university degree. Women were slightly more likely than men to report both post-secondary education (27.6% versus 23.3%) and a university degree (42.7% versus 40.5%). Most respondents were cohabiting. ADL/IADL dependency was reported by 5% and was more common among women (6.3%) than men (3.7%). Most participants reported none or few diseases; only 14.8% reported 4 or more diseases. High symptom burden was reported by 8.6% (women 10%), and very high symptom burden by 5% (women 6.7%).

**Table 2:** Sample characteristics (percent)

N = 3081	N	Percent
**Demographics**		
Women	1519	49.3
Age 60–64	628	20.4
Age 65–69	727	23.6
Age 70–74	668	21.7
Age 75–79	746	24.2
Age 80–85	312	10.1
Lives with others	2273	73.8
**Educational level**		
Primary education or less	197	6.4
Upper-secondary school	807	26.2
Post-secondary studies without university degree	775	25.2
University degree or Ph.D	1267	41.1
**Self-rated health**		
Very poor	18	0.6
Poor	91	3.0
Fair	604	19.6
Good	1606	52.1
Very Good	792	24.7
**Health conditions**		
ADL dependency, yes (≥ 1)	152	4.9
ADL dependency, no	2910	94.4
Number of diseases 0–1	1407	45.7
Number of diseases 2–3	1215	39.4
Number of diseases: 4 or more	456	14.8
**Somatic Symptom Scale (SSS-8)**		
No to minor (0–3)	1115	36.2
Low (4–7)	1016	33.0
Minor (8–11)	508	16.5
High (12–15)	266	8.6
Very high (16–32)	155	5.0

A total of 33 participants were excluded. Of these, 26 were missing age. Nine were missing sex (5 of these were not missing age), and 15 were missing a self-rated health (SRH) score (2 of these were not missing age)

### Vignette ratings

The distribution of the participants’ vignette ratings is presented in Supplementary Table 3. The vignette ratings generally followed the expected ordering pattern at the aggregate level. 55% of participants ranked all four vignettes in the intended order, indicating that a substantial proportion of respondents deviated from the expected vignette ordering.

**Fig. 1:**
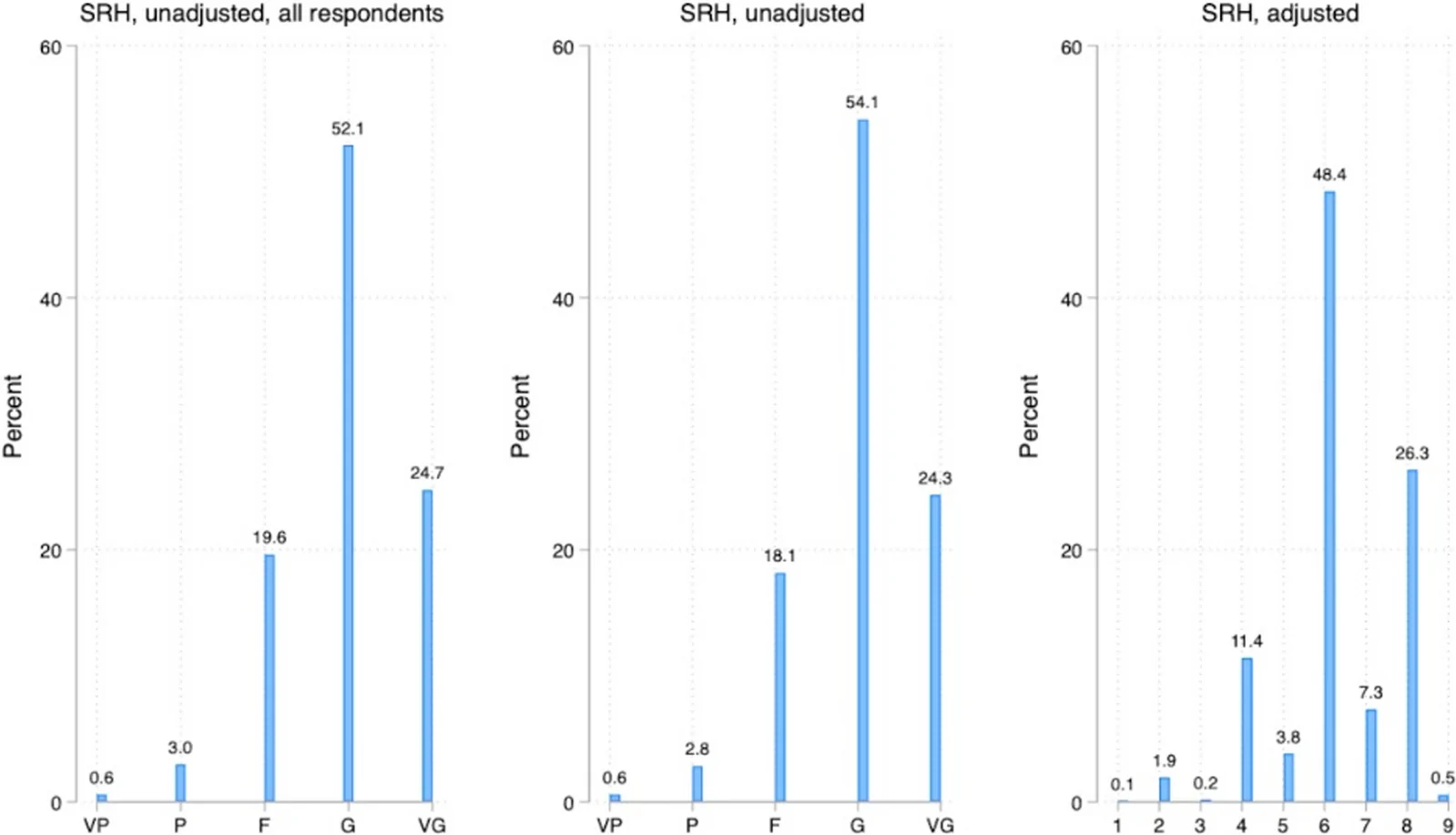
Histograms, unadjusted and adjusted SRH (percent). Comment: In the first histogram, N=3081 (all participants). The second histogram shows the unadjusted SRH for those with correctly ranked vignettes, N=1709. The last histogram presents the non-parametrically adjusted SRH for those with correct vignette rankings, N=1709. The scale has been reversed and ranges from VP=Very poor, P=Poor, F=Fair, G=Good to VG=Very good. Figure 1 was created in STATA

Figure 1 presents histograms of unadjusted and non-parametrically adjusted SRH. The manually adjusted SRH included only participants who correctly ranked the vignettes. The difference in unadjusted SRH between those who ranked the vignettes correctly (mid panel) and all respondents (left panel) was small. The adjusted SRH measure ranges from 1 (very poor) to 9 (very good) compared with the original 1 to 5 scale. Differences in vignette ratings are presented in Supplementary Table 4.

Three groups were significantly less likely to rank the vignettes correctly (Supplementary Table 4): women, individuals with upper secondary education or post-secondary education, and those with ADL/IADL dependency. Women and respondents with post-secondary education or a university degree tend to be more ‘health optimistic’ and appeared to rate the vignettes more positively.

### Comparison between unadjusted and adjusted predictions of SRH—first set of regression analyses

To examine differences between unadjusted SRH and manually (non-parametrically) adjusted SRH among participants who correctly ranked the vignettes, we tested the relationships between the included demographic and health-related predictors and SRH using OPROBIT. The results are presented in Table 3. The most notable difference between the models concerned sex. In the unadjusted model, women reported better self-rated health (SRH) than men. However, this association was attenuated in the adjusted model, and there was no significant difference in SRH ratings between men and women. Similarly, a university degree was positively associated with SRH in the unadjusted model; this association was no longer significant after adjustment. However, additional tests indicated that the education coefficients did not differ significantly between the unadjusted and non-parametically vignette-adjusted models (see Appendix A1). Cohabiting with someone was positively associated with SRH in both the unadjusted and adjusted models. In contrast, ADL/IADL dependency, number of diseases, and symptom burden remained significant in both models.

**Table 3:** Association between demographic and physical factors and SRH, comparison between unadjusted and non-parametric adjusted SRH (OPROBIT models)

	Unadjusted SRH (Scale range 1–5)	**Adjusted SRH** **(Scale range 1–9)**
Women	0.220^***^ (0.059)	0.011 (0.056)
Age (Reference: 60–64 years)		
65–69 years	-0.007 (0.087)	0.008 (0.083)
70–74 years	0.072 (0.089)	0.042 (0.085)
75–79 years	0.048 (0.088)	-0.010 (0.084)
80–85 years	0.037 (0.115)	-0.019 (0.111)
Education (Reference: primary school or less)		
Upper-secondary school	0.216 (0.122)	0.094 (0.118)
Post-secondary studies	0.172 (0.122)	0.035 (0.118)
University degree/Ph.D	0.239^*^ (0.116)	0.104 (0.112)
Live with others	0.175^**^ (0.067)	0.254^***^ (0.065)
ADL	-1.121^***^ (0.154)	-1.028^***^ (0.152)
Disease burden (0–1 reference)		
2–3	-0.572^***^ (0.066)	-0.541^***^ (0.063)
4 or more	-0.731^***^ (0.094)	-0.742^***^ (0.091)
Somatic Symptoms Scale (1–5)	-0.564^***^ (0.030)	-0.500^***^ (0.029)
Cut 1	-4.800^***^ **(0.234)**	-3.990^***^ **(0.186)**
Cut 2	-3.716^***^ **(0.183)**	-3.933^***^ **(0.184)**
Cut 3	-2.141^***^ **(0.160)**	-2.589^***^ **(0.158)**
Cut 4	-0.151 (0.151)	-2.354^***^ **(0.156)**
Cut 5		-0.571^***^ **(0.146)**
Cut 6		-0.313^*^ **(0.146)**
Cut 7		1.928^***^ **(0.191)**
R2 Adjusted		
Observations	1641	1641
LL	-1443.675	-1915.371

Significance levels: *p < 0.05, ^**^ *p* < 0.01, ^***^ *p* < 0.001

### Comparison between unadjusted and adjusted predictions of SRH—second set of regression analyses

To address potential reporting heterogeneity and systematic differences in response styles, we compared a CHOPIT model accounting for differential response thresholds with an unadjusted OPROBIT model, both including all respondents. The results were presented in Table 4 and predicted probabilities calculated from the OPROBIT model are presented in Supplementary Table 5. Several associations changed after accounting for reporting heterogeneity. Women reported better SRH in the unadjusted OPROBIT model, but this association reversed in the CHOPIT model, indicating worse latent health among women after accounting for differences in response thresholds. The threshold estimates also indicated sex-related differences in the use of the SRH response scale: women were more likely than men to rate their health as “poor” rather than “very poor”.

**Table 4:** Unadjusted (OPROBIT) and adjusted predictions of SRH (CHOPIT)

	**OPROBIT** **β**	**SE**	**CHOPIT** **β**	**SE**
Woman	0.273^***^	**(0.044)**	-0.247***	**(0.053)**
Age (Reference: 60–64 years)				
65–69 years	0.073	(0.064)	0.077	(0.077)
70–74 years	0.144^*^	**(0.066)**	0.082	(0.079)
75–79 years	0.030	(0.065)	0.124	(0.078)
80–85 years	0.038	(0.084)	0.080	(0.101)
Education (Reference: Primary school or less)				
Upper-secondary school	0.131	(0.094)	0.024	(0.112)
Post-secondary studies	0.224^*^	**(0.094)**	0.073	(0.113)
University degree	0.295^**^	**(0.090)**	-0.004	(0.109)
Live with others	0.158^**^	**(0.049)**	0.208***	**(0.059)**
ADL	-1.122^***^	**(0.104)**	-1.327***	**(0.124)**
Physical disease burden (0–1 reference)				
2–3	-0.504^***^	**(0.048)**	-0.501***	**(0.058)**
4 or more	-0.675^***^	**(0.070)**	-0.742***	**(0.086)**
Somatic Symptoms Scale	-0.529^***^	**(0.022)**	-0.550***	**(0.026)**
**Threshold 1 (very poor–poor)**				
Woman			-0.545***	**(0.048)**
**65–69 **years			-0.182**	**(0.066)**
**70–74 **years			-0.247***	**(0.070)**
**75–79 **years			-0.210**	**(0.068)**
**80–85 **years			-0.374***	**(0.091)**
Upper-secondary school			-0.107	(0.099)
Post-secondary studies			-0.082	(0.099)
University degree			-0.093	(0.094)
Live with others			0.036	(0.053)
ADL			0.169	(0.106)
Disease burden 2–3			0.013	(0.050)
Disease burden 4 or more			0.081	(0.076)
Somatic symptoms scale			-0.004	(0.021)
Constant	-4.537^***^	**(0.171)**	-5.468***	**(0.208)**
**Threshold 2 (poor–fair)**				
Woman			-0.002	(0.028)
65–69 years			0.057	(0.041)
70–74 years			0.040	(0.043)
75–79 years			0.041	(0.042)
80–85 years			0.103	(0.054)
Upper-secondary school			0.063	(0.062)
Post-secondary studies			0.060	(0.062)
University degree			0.021	(0.059)
Live with others			-0.001	(0.032)
**ADL**			-0167*	**(0.070)**
Disease burden 2–3			-0.017	(0.030)
Disease burden 4 or more			-0.083	(0.047)
Somatic symptoms scale			-0.010	(0.013)
Constant	-3.491^***^	**(0.135)**	0.672***	**(0.078)**
**Threshold 3 (fair–good)**				
Woman			0.039	0.029
65–69 years			0.017	0.043
70–74 years			0.028	0.044
75–79 years			0.089*	**0.043**
80–85 years			0.095	0.054
Upper-secondary school			-0.087	0.057
Post-secondary studies			-0.086	0.057
University degree			-0.138*	**0.054**
Live with others			0.002	0.032
ADL			0.051	0.059
Disease burden 2–3			0.006	0.032
Disease burden 4 or more			-0.009	0.046
Somatic symptoms scale			0.008	0.013
Constant	-1.903^***^	**(0.120)**	0.534***	**0.075**
**Threshold 4 (good–very good)**				
Woman			-0.031	0.025
65–69 years			0.053	0.037
70–74 years			0.067	0.038
75–79 years			0.088	0.038
80–85 years			0.054	0.049
Upper-secondary school			-0.006	0.054
Post-secondary studies			-0.080	0.054
University degree			-0.047	0.051
Live with others			-0.042	0.052
ADL			0.022	0.066
Disease burden 2–3			-0.016	0.027
Disease burden 4 or more			0.008	0.041
Somatic symptoms scale			-0.001	0.012
Constant	-0.030	(0.115)	0.551***	**(0.069)**
Observations	2924		SRH 2894 Vignettes 2896	
Log likelihood	-2656.193		11,914	

The CHOPIT model includes respondents who contribute to either the self-assessment equation or the vignette-based threshold equations. In our data, 2,896 individuals provided at least one vignette rating and, therefore, contributed to the estimation of reporting thresholds, while 2,894 individuals provided a self-rated health response and contributed to the latent-health equation. The overall model sample, therefore, consists of 2,896 unique respondents

Significance levels: *p < 0.05, ^**^ *p* < 0.01, ^***^ *p* < 0.001.

Higher education (post-secondary studies and university degree) was positively associated with SRH in the unadjusted OPROBIT model; however, the associations between educational attainment and SRH were no longer significant after adjustment in the CHOPIT model. At the same time, the threshold estimates indicated education-related differences in the use of the response categories. In particular, the threshold pattern suggests that respondents with a university degree were more likely to rate their own health as “good” rather than “fair” at comparable levels of latent health. Taken together, this pattern is consistent with education-related differential item functioning: part of the observed educational gradient in conventional SRH appears to reflect differences in how respondents use the response scale rather than differences in latent health.

The positive association between age 70–74 and SRH observed in the OPROBIT model was also no longer significant after adjustment. For those aged 75–79, the threshold between “fair” and “good” was significant, suggesting that at the same latent-health level, this age group was less likely to rate their health as good rather than fair. Several associations remained stable across both models. Living with others continued to be positively associated with SRH, while the ADL/IADL dependency, number of reported diseases, and symptom burden remained negatively associated with SRH. In addition, the significant negative threshold between “poor” and “fair” SRH for individuals with ADL/IADL dependency indicates that, at the same latent-health status, these respondents were more likely to rate their health as fair rather than poor.

## Discussion

This study investigated the association between demographic and health factors and SRH, both unadjusted and adjusted using anchoring vignettes in a Swedish population of older adults aged 60–85. By applying the anchoring vignette method, we moved beyond simply documenting differences in reporting to examining their underlying causes. Specifically, we sought to determine whether observed differences in SRH reflected genuine health disparities (which would persist after adjustment) or DIF, that is, varying norms and thresholds for evaluating and reporting health, which would be revealed by discrepancies between unadjusted and adjusted models. To address this, we conducted two sets of analyses. First, we used OPROBIT models to compare unadjusted and manually adjusted SRH among participants who correctly ranked the vignettes. Second, we applied OPROBIT and CHOPIT models (King et al. 2003; Grol-Prokopczyk et al. 2011) to compare unadjusted (OPROBIT) and adjusted (CHOPIT) SRH among all respondents with valid data. The advantage of the CHOPIT approach is that it allows reporting heterogeneity to be modelled without relying on strict ranking assumptions, thereby permitting the retention of all observations, including those from participants with inconsistent vignette rankings.

Our results revealed a distinct pattern of sex differences. In the unadjusted analyses, women reported better SRH than men. This difference disappeared in the first adjusted model and was reversed in the more comprehensive CHOPIT model, in which women had worse latent SRH than men. This reversal suggests that the initial difference between men and women may reflect DIF and differing reporting norms rather than genuine differences in latent health. The adjusted results align with previous research indicating that women often report worse SRH than men (Benyamini et al. 2000; Feenstra et al. 2020; Thörnqvist et al. 2025; Zajacova et al. 2017). The initial, more positive rating reported by women in our sample may be explained by both socioeconomic and psychological factors. Women in this study exhibited exceptionally high educational attainment, a factor strongly associated with better SRH, which may have inflated their unadjusted SRH ratings. Prior research suggests that women may display greater "health optimism" in survey settings, potentially reflecting stronger coping strategies or a tendency to downplay health complaints (Ping & Oshio 2023; Pinillos-Franco & García-Prieto 2017; Schaap et al. 2024). While such psychological resilience is beneficial, it may introduce systematic bias into self-reported health measures. Age followed a similar pattern in the second set of analyses. The significant association between being aged 70–74 and better SRH observed in the unadjusted model was no longer statistically significant after adjustment. This indicates that, once chronic diseases, level of physical symptoms, and ADL/IADL dependency were accounted for, there was no longer evidence of better latent health among individuals aged 70–74 compared with the reference age group. In other words, the unadjusted results may partly reflect differences in response-scale use among individuals aged 70–74, rather than superior latent-health status. The positive association between higher education and SRH was no longer statistically significant in the CHOPIT model. The attenuation of the education coefficient is consistent with education-related DIF. The observed threshold differences suggest that respondents with university degree may use different criteria when rating their own health, especially around the distinction between “fair” and “good” health. The CHOPIT model identifies education-related variation in the fair–good response threshold, and once these differences are accounted for, the estimated education effect on latent health is substantially reduced and no longer statistically significant. A similar mechanism may explain the attenuation in the non-parametrically adjusted measure, since respondents' self-ratings are rescaled relative to their own vignette ratings. However, that comparison also restricts the sample to respondents whose vignette rankings are strictly ordered, so the difference from the full-sample model reflects both vignette adjustment and sample composition.

In contrast, symptom burden, number of diseases, and dependence in ADL/IADL emerged as robust and consistent predictors of worse SRH across both adjusted and unadjusted models. As these factors are included in all analyses, they represent key dimensions of objective health status accounted for throughout the study. These findings underscore that SRH captures the lived experience of illness and functional limitations that resonate across different reporting styles and subjective thresholds. Although objective indicators are valuable, they typically do not reflect the internal assessment that only the individual can make. In this sense, SRH captures aspects of health that remain inaccessible to external observation or narrow clinical metrics. Taken together, our results indicate that some predictors are robust across different modelling approaches, suggesting that conventional unadjusted SRH remains a meaningful measure for further research. Nevertheless, caution is warranted to avoid underestimating or overestimating the relative importance of certain factors, as residual differences may reflect both reporting behaviour and aspects of health not captured by the included objective measures. Moreover, reporting behaviour may mask or distort underlying differences in SRH.

Our findings can also be interpreted in relation to the perspective that the importance of different determinants of SRH may change across the life course (Galenkamp et al. 2013). Although our analyses are cross-sectional and, therefore, do not capture change over time, they are age-adjusted. The strong associations observed between SRH, and the included physical health factors suggest that these dimensions are central to how older adults evaluate their health.

Studying SRH through anchoring vignettes also raises broader epistemological questions about health measurement—namely, what does SRH truly represent? Is it an indisputable expression of personal experience, or can individuals ‘misinterpret’ their own health? The rationale for using anchoring vignettes is not that individuals are mistaken about their health, but rather that their self-reports are filtered through distinct cultural, social, and psychological lenses, which may impede direct comparison. Anchoring vignettes do not discard subjective health experiences; instead, they refine them into a more comparable metric.

However, applying the anchoring vignette approach requires specific implementation strategies in survey design as well as complex statistical modelling. One of the key strengths of SRH lies in its simplicity and strong predictive power for morbidity, healthcare utilisation, and mortality. The anchoring vignette method, therefore, sacrifices methodological simplicity in exchange for greater precision and fairness in group comparisons. Although more research is needed, one pragmatic and context-dependent approach may be that in studies focused on prediction (e.g. mortality risk), unadjusted SRH may remain sufficient, whereas in studies focused on comparison (e.g. comparing health levels across sex, cultures, or countries), adjusted SRH should be used to avoid biased conclusions.

### Strengths and limitations

To provide a comprehensive understanding of the study’s contributions, its strengths and limitations are considered below. A key strength of this study is the inclusion of a broad age range of older adults (60–85 years), together with an even distribution of men and women. A further strength is the strict adherence to all methodological steps for anchoring vignette studies outlined by King and Wand (2007), which enhances the validity of the findings. Several limitations should nonetheless be acknowledged. First, the sample's educational level was higher than in the general Swedish population, with more than 40% of participants holding a university degree. This may influence the generalisability of the findings, since higher education can be associated with better SRH and may influence how people interpret and respond to health questions. Second, although the CHOPIT model retains respondents with inconsistent vignette rankings, only 55% ranked all four vignettes in the expected order. This mainly concerns the non-parametric adjustment. However, the variation in vignette ordering may still reflect differences in vignette interpretation or health concepts and should be considered when interpreting the findings. Third, the survey was administered online, requiring access to a computer, tablet, or smartphone, as well as sufficient cognitive and visual abilities—factors that may further constrain generalisability. Nevertheless, we argue that the knowledge gained from this study is of general relevance, as it concerns the usefulness and suitability of anchoring vignettes within an older Swedish population. Fourth, the sample was relatively homogeneous, which may reduce the extent of DIF compared with more diverse or cross-national samples. In particular, older adults with an immigrant background were not represented, a group that is hard to reach for various reasons. Given the growing ageing and increasingly diverse ageing population, future research should aim to include older adults from a wider range of cultural backgrounds.

## Conclusion

This study demonstrates that anchoring vignettes are a suitable tool for evaluating SRH in later life. By applying anchoring vignettes when examining SRH, this study provides a more comparable assessment of subjective health and highlights the importance of considering reporting behaviour when interpreting demographic differences in SRH. After accounting for reporting differences, the factors most robustly associated with SRH in later life were related to physical health and functional status. The association between education and SRH was attenuated and no longer statistically significant after adjustment, a pattern consistent with education-related differences in reporting behaviour. In the unadjusted analyses, women reported better SRH than men. However, this pattern reversed after adjustment, revealing poorer SRH among women at equivalent levels of latent health. Together, these results highlight the value of anchoring vignettes in clarifying subjective health differences between groups and support the use of a complementary tool in the analysis of SRH in later life. More broadly, this study demonstrates that adjusting SRH using anchoring vignettes can meaningfully alter observed demographic gradients in later-life health assessments. In particular, differences by sex and education may partly reflect variation in how respondents interpret and apply response categories. These findings underscore the importance of accounting for reporting heterogeneity when using subjective health measures in ageing research. Incorporating anchoring vignette approaches may, therefore, improve the comparability and interpretation of SRH in studies of older populations.

## Supplementary Information

Below is the link to the electronic supplementary material.

Supplementary Material 1.

## Data Availability

Data are available upon request to the corresponding author, Karolina Thune, at karolina.thornqvist@gu.se. This study was not preregistered.
