# Supplementary material for: Predictors of self-rated health in older adults: insights from an anchoring vignettes study
Source: Eur J Ageing. 2026 Oct 6;23(1):39. doi: 10.1007/s10433-026-00952-7 (PMC13642853; doi:10.1007/s10433-026-00952-7)
Supplement: Supplementary file 1 [file 10433_2026_952_MOESM1_ESM.docx]

**Predictors of Self-Rated Health in Older Adults: Insights from an Anchoring Vignettes Study**

Karolina Thune, MA^1*^, Hanna Falk Erhag, PhD^1,2^ , Anne Ingeborg Berg, PhD^3^,

Lena Johansson, PhD^1,5^, Maria Solevid, PhD^6^

^1^Institute for Health and Care Science, Sahlgrenska Academy, University of Gothenburg, Gothenburg, Sweden.

*Corresponding author: Karolina Thune, karolina.thornqvist@gu.se, Box 457, 40530 Gothenburg, Sweden

Submitting to European Journal of Ageing

**Supplementary Table 1. Distribution of invited participants**

|  | N | Percent |
| --- | --- | --- |
| Women, 60-69 years | 963 | 22.40 |
| Women, 70-74 years | 449 | 10.44 |
| Women, 75-85 years | 796 | 18.51 |
| Men, 60-69 years | 962 | 22.37 |
| Men, 70-74 years | 427 | 9.93 |
| Men, 75-85 years | 703 | 16.35 |
| Total | 4300 | 100.00 |
|  |  |  |

| Supplementary Table 2. ADL/iADL Dependency (Swedish and English translation) | |
| --- | --- |
| Swedish: Är du i behov av hjälp med någon av följande aktiviteter i din vardag? (Det avser om du behöver hjälp av annan person för att utföra dessa aktiviteter i din vardag. Inte om du är i behov av hjälpmedel). | English: Do you need help with any of the following activities in your daily life? (This refers to whether you need help from another person to perform these activities in your daily life. It does not refer to whether you need assistive devices). |
| Städning | Cleaning |
| Handla | Shopping |
| Transport | Transport |
| Matlagning | Cooking |
| Badning/Dusch | Bathing/Showering |
| På/avklädning | Dressing/Undressing |
| Toalett | Toilet |
| Förflyttning (säng-stol) | Moving (bed-chair) |
| Födointag (äta) | Eating |
| Telefonera | Telephoning |
| Sköta hushåll | Household chores |
| Tvätta | Washing |
| Sköta egen medicinering | Taking medication |
| Sköta egen ekonomi | Managing personal economy |

**Supplementary Table 3. Vignette ratings (percent)**

| **Vignette ratings** | **Vignette 1 Very good/good SRH** | **Vignette 2 Good/Fair SRH** | **Vignette 3 Fair/Poor SRH** | **Vignette 4 Poor/ Very Poor SRH** |
| --- | --- | --- | --- | --- |
| **N** | **3056** | **3041** | **3046** | **3039** |
| *Mean* | *4.8* | *3.9* | *2.6* | *1.7* |
| Very good | **84.8** | 17.0 | 0.1 | 0.0 |
| Good | 13.7 | **59.6** | 5.9 | 0.3 |
| Fair | 1.2 | 21.4 | **54.0** | 4.2 |
| Poor | 0.2 | 1.7 | 38.7 | **58.8** |
| Very poor | 0.1 | 0.2 | 1.4 | 36.7 |

**Supplementary Table 4. The association between sociodemographic and health variables and vignette ratings (unstandardized b coefficients, standard errors in parentheses),**

|  | **Dependent variable: Rating of health status in vignettes**  **(1-5, 1=Very bad, 5=Very good)** | | | | |
| --- | --- | --- | --- | --- | --- |
|  | Vignette 1  (Very good/good health) | Vignette 2  (Good-fair health) | Vignette 3  (Fair-bad health) | Vignette 4  (Bad/very bad health) | Index of vignette ratings |
| Self-rated health, (1-5, 1=Very bad, 5=Very good) | **0.063^***^ (0.013)** | **0.115^***^ (0.020)** | **0.065^***^ (0.018)** | 0.023 (0.017) | **0.055^***^ (0.008)** |
| Woman | **0.109^***^ (0.016)** | **0.350^***^ (0.025)** | **0.276^***^ (0.023)** | **0.196^***^ (0.021)** | **0.135^***^ (0.011)** |
| Age (Reference: 60-64 yrs) |  |  |  |  |  |
| 65-69 yrs | -0.009 (0.023) | 0.031 (0.036) | 0.028 (0.033) | 0.052 (0.031) | -0.000 (0.016) |
| 70-74 yrs | 0.010 (0.024) | 0.009 (0.037) | **0.086^*^ (0.034)** | **0.116^***^ (0.032)** | -0.003 (0.016) |
| 75-79 yrs | -0.044 (0.024) | -0.021 (0.037) | 0.032 (0.034) | **0.095^**^ (0.032)** | **-0.032^*^ (0.016)** |
| 80-85 yrs | -0.035 (0.031) | 0.013 (0.048) | **0.098^*^ (0.044)** | **0.128^**^ (0.041)** | -0.013 (0.021) |
| Education (Reference: Primary school or less) |  |  |  |  |  |
| Upper-secondary school | 0.041 (0.035) | **0.143^**^ (0.053)** | -0.031 (0.049) | -0.013 (0.047) | 0.042 (0.023) |
| Post-secondary studies | 0.063 (0.035) | **0.156^**^ (0.054)** | -0.037 (0.050) | -0.025 (0.047) | **0.052^*^ (0.023)** |
| University degree | **0.122^***^ (0.034)** | **0.217^***^ (0.052)** | 0.020 (0.048) | -0.034 (0.045) | **0.098^***^ (0.022)** |
| Lives with other/s | -0.017 (0.018) | 0.003 (0.028) | -0.028 (0.026) | **-0.053^*^ (0.024)** | 0.003 (0.012) |
| ADL | **-0.082^*^ (0.040)** | 0.117 (0.061) | **0.140^*^ (0.056)** | 0.065 (0.053) | 0.028 (0.026) |
| Disease burden (0-1 reference) |  |  |  |  |  |
| 2-3 | 0.022 (0.018) | **0.057^*^ (0.027)** | 0.013 (0.025) | -0.032 (0.024) | **0.031^**^ (0.012)** |
| 4 or more | **0.055^*^ (0.026)** | **0.122^**^ (0.040)** | 0.042 (0.037) | -0.032 (0.035) | **0.063^***^ (0.017)** |
| Somatic symptoms scale (1-5) | 0.012 (0.009) | **0.045^***^ (0.013)** | **0.036^**^ (0.012)** | 0.012 (0.011) | **0.020^***^ (0.006)** |
| Constant | 4.439^***^ (0.071) | 2.975^***^ (0.109) | 2.148^***^ (0.101) | 1.473^***^ (0.095) | 3.522^***^ (0.047) |
| Observations | 2894 | 2894 | 2894 | 2894 | 2894 |
| Adjusted *R*^2^ | 0.040 | 0.094 | 0.066 | 0.037 | 0.087 |

Significance levels: **p* < 0.05, ^**^ *p* < 0.01, ^***^ *p* < 0.001

**Supplementary Table 5. Predicted probabilities for different outcomes of SRH, significant coefficients (all other variables held at their means)**

|  | **Very bad** | **Bad** | **Fair** | **Good** | **Very good** |
| --- | --- | --- | --- | --- | --- |
| Man (ref) | .00 | .01 | .20 | .65 | .15 |
| Woman | .00 | .00 | .13 | .65 | .22 |
|  |  |  |  |  |  |
| Age 60-54 (ref) | .00 | .01 | .18 | .65 | .16 |
| Age 70-74 | .00 | .00 | .15 | .65 | .20 |
|  |  |  |  |  |  |
| Primary school or less (ref) | .00 | .01 | .22 | .64 | .13 |
| Post-secondary studies | .00 | .01 | .16 | .65 | .18 |
| University degree/Ph.D | .00 | .00 | .15 | .65 | .20 |
|  |  |  |  |  |  |
| Cohabitation No (ref) | .00 | .01 | .19 | .65 | .15 |
| Cohabitation Yes | .00 | .01 | .16 | .65 | .19 |
|  |  |  |  |  |  |
| ADL No (ref) | .00 | .01 | .15 | .65 | .19 |
| ADL Yes | .00 | .07 | .48 | .43 | .02 |
|  |  |  |  |  |  |
| Disease burden 0-1 | .00 | .00 | .10 | .63 | .27 |
| Disease burden 2-3 | .00 | .01 | .22 | .64 | .13 |
| Disease burden 4 or more | .00 | .02 | .27 | .62 | .10 |
|  |  |  |  |  |  |
| Physical symptoms score 0-3 | .00 | .00 | .06 | .57 | .37 |
| Physical symptoms score 4-7 | .00 | .00 | .15 | .65 | .20 |
| Physical symptoms score 8-11 | .00 | .02 | .29 | .60 | .08 |
| Physical symptoms score 12-15 | .00 | .06 | .46 | .45 | .03 |
| Physical symptoms score 16-32 | .02 | .14 | .56 | .28 | .01 |

 Notes: Predicted probabilities were calculated using Stata’s *margins* command, with all other variables in the model held at their means.

**Appendix A1. Supplementary analysis: Understanding the association between education and SRH**

***Formal test of difference between the education gradient in the manually unadjusted and adjusted SRH models***

To assess whether the education coefficients differed significantly between the unadjusted and the manually (non-parametric) vignette-adjusted SRH models, we conducted two supplementary tests.

First, we conducted Wald tests after seemingly unrelated estimation of the ordered probit models using Stata’s suest command. This test showed no statistically significant difference in the education coefficients across the unadjusted and manually vignette-adjusted models, χ²(3)=4.30, p=.231. However, this result should be interpreted with caution, as ordered probit coefficients are estimated on latent scales and the two outcomes have different numbers of ordinal categories (5 categories in the unadjusted measure and 9 categories in the adjusted measure).

Second, we estimated a stacked linear model using standardized (z-scored) versions of the unadjusted and adjusted SRH outcomes using Stata’s testparm command. This approach provides a less scale-dependent comparison than direct comparisons of ordered probit coefficients, because both dependent variables are placed on the same standard deviation metric before the coefficient differences are tested. The model included interactions between an indicator for vignette-adjusted SRH and the covariates, with standard errors clustered at the respondent level. The joint test for the education interactions was also not statistically significant, F(3,1640)=1.28, p=.279.

Taken together, these two tests indicate that the education coefficients do not differ significantly between the unadjusted and the manually adjusted SRH models. Thus, although the association between education and SRH was reduced and no longer statistically significant after manual adjustment, this reduction was not itself statistically significant.

However, results should be treated with some caution as 1) only half of the respondents are included in these models (the non-parametric, manually, adjusted SRH measure requires correct vignette rankings), and 2) comparisons across the two models, as indicated above, are less straightforward as the scale of the dependent variable differ.

***Reaching conclusions about education and SRH***
We base our conclusion about the insignificant association between education and (latent) SRH on the following chain of evidence, and most importantly, we rely on the OPROBIT and CHOPIT models on the full sample (Table 4 in main text).

1) Higher education is associated with better unadjusted self-rated health (SRH) in the full sample (Table 4 in Main text, column OPROBIT)
2) Higher education is associated with more favourable ratings of identical vignette health states in the full sample (Supplementary Table 4).
3) The CHOPIT model in the full sample indicates education-related variation in response thresholds, especially between Fair and Good health (Table 4 in Main text, column CHOPIT). This pattern is consistent with differential item functioning (DIF) by education that we see traces of already in the assessments of the vignettes (point 2 above).
4) Once this reporting heterogeneity is taken into account, the association between education and latent health is reduced to approximately zero.
